# Combining an SSRI with an anticonvulsant in depressed patients with dysphoric mood: an open study

**DOI:** 10.1186/1745-0179-3-3

**Published:** 2007-02-08

**Authors:** Massimo Pasquini, Angelo Picardi, Azzurra Speca, Valerio Orlandi, Lorenzo Tarsitani, Pierluigi Morosini, Isabella Cascavilla, Massimo Biondi

**Affiliations:** 1 Department of Psychiatric Science and Psychological Medicine, University "La Sapienza" of Rome, Viale dell'Università 30, 00185 Rome, Italy; 2 National Center of Epidemiology and Health Surveillance and Promotion, Italian National Institute of Health, Viale Regina Elena 299, 00161 Rome, Italy

## Abstract

**Background:**

Several patients with unipolar depression present with prominent dysphoric mood. We aimed at examining the effectiveness of the combination of an SSRI with an anticonvulsant in such patients.

**Methods:**

Thirty-five newly admitted outpatients with substantial anger, irritability, aggressiveness or hostility who were diagnosed a DSM-IV unipolar depressive disorder were rated on the Hamilton Depression Rating Scale (HDRS), the Clinical Global Improvement (CGI) scale, and a scale for the rapid dimensional assessment (SVARAD), were prescribed an SSRI and an anticonvulsant (usually valproate), and were followed up for 12 weeks. Repeated measures analysis of variance was used to test for within-subject changes in scale scores over time.

**Results:**

Thirty-two and 23 patients attended the follow-up visits 4 and 12 weeks later, respectively. Significant decreases (p < .001) were observed in HDRS total score, HDRS and SVARAD anxiety factors, HDRS and SVARAD core depression factors, and SVARAD anger/irritability factor. Adjusting for age or gender did not change the results. Most patients (82%) were rated as improved or much improved on the CGI.

**Conclusion:**

Although our study has several limitations, we observed a remarkable improvement in most unipolar depressed outpatients with dysphoric mood treated with an SSRI and an anticonvulsant. The effectiveness of anticonvulsants might be linked to their action on symptoms of aggression and behavioural activation.

## Background

Since many decades, psychopathologists have pointed up that nonmelancholic and even melancholic depressions often entail anger [1]. However, until relatively recently, there were a scarcity of research findings related to this issue. This might partly be ascribed to the use of specific rating scales for depression, which tend to put all depressions 'in the same basket'[2] and have been the object of some criticisms [3-6]. More recently, the use of comprehensive psychopathological rating scales exploring a wide range of symptoms has enabled several researchers to document substantial levels of anger, irritability, hostility and aggressiveness in a sizable portion of patients with unipolar depression [7-10]. Indeed, anger and aggression are prominent in depressed patients to a degree that may rival that of depression and anxiety [11], and anger is higher among patients with depression compared with patients with anxiety or somatoform disorders [12,13]. These patients have particular needs that should be addressed, and many clinicians share the opinion that antidepressant medication alone may not be fully adequate. A treatment approach based on functional/dimensional psychopathology [20,21], might be useful to optimise pharmacological treatment for these patients. In accordance with the hypothesised implication of serotonergic pathways in the brain in aggression [16-18], many authors have suggested that SSRIs might be first choice antidepressants in patients with depression with hostility [16,19,20]. Other drugs able to reduce aggressiveness through other biochemical mechanisms, such as some anticonvulsants affecting the GABAergic and glutammatergic systems, might also be useful in these patients. However, to our knowledge, no trial has tested the efficacy of valproate or other anticonvulsants in the treatment of depression with dysphoric mood.

In this paper, we report the results of a pilot study where we examined the effectiveness of the combination of an SSRI with an anticonvulsant in patients with unipolar depression with prominent anger, irritability, hostility, and aggressiveness.

## Methods

### Patients

The study was performed between March 2002 and May 2004 at the Psychopharmacology Outpatient Clinic of the Department of Psychiatric Sciences and Psychological Medicine of the 'La Sapienza' University of Rome. All patients newly admitted on predetermined weekdays were considered for enrolment in the study, according to the following inclusion criteria: a) at least 18 years of age; b) current diagnosis of a DSM-IV Depressive Disorder, with the exception of bipolar spectrum conditions (Bipolar Disorder or Cyclothymic Disorder); c) presence of notable anger, aggressiveness or hostility as attested by a rating of 1 or higher on the corresponding item of the SVARAD (see below); d) no comorbid Cluster A Personality disorder or Borderline Personality Disorder; e) no treatment with antidepressant drugs in the preceding 2 months; f) absence of severe physical illness. A total of 35 consecutive patients meeting these criteria were included in the study. Their demographic and clinical characteristics are summarised in Table 1.

**Table 1:** Demographic and clinical characteristics of patients

Gender		12 males *(34%)* 23 females *(66%)*
Age (mean ± SD)		44.6 ± 11.7
Main diagnosis		Major Depressive Disorder 27 *(77%)* Dysthymic Disorder 4 *(11%)* Depressive Disorder NOS 3 *(9%)* Adjustment Disorder with Depressed Mood 1 *(3%)*

Psychiatric comorbidity		No comorbid axis I or II disorder 27 *(77%)* Panic Disorder 2 *(6%)* Generalised Anxiety Disorder 1 *(3%)* Bulimia Nervosa 1 *(3%)* Eating Disorder NOS 1 *(3%)* Conversion Disorder 1 *(3%)* Histrionic Personality Disorder 1 *(3%)* Dependent Personality Disorder 1 *(3%)*

HDRS baseline score (mean ± SD)		17.3 ± 3.3

Antidepressant drug prescribed	Paroxetine	11 (31%) (mean dose 24 mg/die, range 20–30)
	Fluvoxamine	10 *(29%)* (mean dose 125 mg/die, range 100–250)
	Citalopram	10 *(29%)* (mean dose 29 mg/die, range 20–40)
	Fluoxetine	2 *(6%)* (both patients on 20 mg/die)
	Sertraline	2 *(6%)* (mean dose 125 mg/die, range 100–150)

Anticonvulsant prescribed	Valproate	26 *(74%) *(mean dose 438 mg/die, range 200–1000)
	Carbamazepine	3 *(9%)* (mean dose 550 mg/die, range 450–600)
	Gabapentin	6 *(17%)* (mean dose 517 mg/die, range 300–600)

**Legend**: HDRS = Hamilton Depression Rating Scale

### Procedure

At admission, all patients underwent a careful psychiatric examination lasting about 90 minutes, and then were diagnosed according to DSM-IV criteria. All diagnoses were confirmed by a faculty psychiatrist with more than 25 years of clinical experience who reviewed all clinical records. Also, patients were rated on the 17-item Hamilton Depression Rating Scale (HDRS) [21], and the SVARAD. The SVARAD (acronym for the Italian name 'Scala per la VAlutazione RApida Dimensionale', i.e. scale for rapid dimensional assessment) is a 10-item instrument specifically developed for the rapid assessment of the main psychopathological dimensions [22]. A validation study has documented interrater reliability, content validity, and criterion validity [22]. The items explore the following dimensions: 1) apprehension/fear; 2) sadness/demoralization; 3) anger/aggressiveness; 4) obsessiveness; 5) apathy; 6) impulsivity; 7) reality distortion; 8) thought disorganization; 9) somatic preoccupation/somatization; 10) activation. Item 3 is defined as follows: irritation, anger, resentment; irritability, litigiousness, hostility; verbal or physical violence. The items are rated on a 5-point scale ranging from 0 to 4, with higher scores indicating greater severity. All raters had great familiarity with this instrument and had been trained in its proper use.

All patients were prescribed a Selective Serotonin Reuptake Inhibitor (SSRI) and an anticonvulsant effective on symptoms of aggression and behavioural activation, and were instructed to come back to the outpatients clinic at regular intervals for follow-up visits. For each patient, medication was chosen by the treating psychiatrist openly without any fixed schemata and according to clinical impression. Drug dosages were titrated individually. Details about drug treatment are reported in Table 1. After 4 and 12 weeks, patients were rated again on the HDRS and the SVARAD. In addition, they were rated on the Global Improvement item of the Clinical Global Improvement (CGI) scale [27].

### Data reduction and statistical analysis

There is ample evidence that the HDRS is not unidimensional [2]. While the number of factors identified ranges widely, there is strong support for the presence a 'core depression' factor and an 'anxiety' factor, as well as for a sleep disturbance factor. According to the results of a recent confirmatory factor analysis [23], which shared many similarities with a previous study by our group [24], the 'core depression' factor is loaded by items 1, 7 and 8 (depressed mood, work and activities, retardation), while the 'anxiety' factor is loaded by items 10, 11, and 15 (psychic anxiety, somatic anxiety, hypochondriasis). Hence, for the HDRS, we calculated the scores on these two subsets of items in addition to the total score for each time point. Also, given the potential risk of deliberate self-harm in these patients we analysed separately the HDRS item on suicide.

As regards the SVARAD, two studies on depressed patients [10,25] have consistently shown three factors, namely an "anger/irritability" dimension (anger/aggressiveness, impulsivity, and activation), a "pure depression" dimension (sadness/demoralization, apathy), and an "anxiety" dimension (apprehension/fear, somatic preoccupation/somatization). Therefore, scores on these three factors were calculated for each time point.

Repeated measures analysis of variance (ANOVA) was used to test for within-subject changes in scores on the HDRS and the SVARAD over time. Greenhouse-Geisser correction was used to adjust degrees of freedom for all F tests if Mauchly's test of sphericity indicated heterogeneity of covariances. All ANOVAs yielding significant results were performed again adjusting for gender and age (≤ 45 *vs*. > 45 years) in two separate models. Given that the number of patients receiving some drugs was too small to build ANOVA models adjusting for the prescribed antidepressant or anticonvulsant, the findings were stratified by medication type in order to visually inspect whether the changes over time in outcome measures were consistent across strata. Change in scores on the HDRS suicide item were tested with Wilcoxon's nonparametric test.

## Results

**Figure 1:**
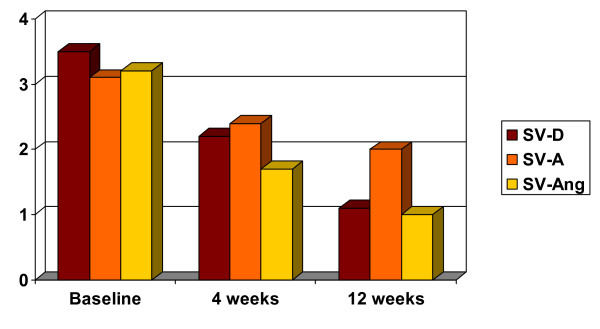
Significant decrease of SVARAD from baseline to 4 and 12 weeks. SV-D = SVARAD-Depresseive Factor; SV-A = SVARAD-Anxiety Factor; SV-Ang = SVARAD-Anger/irritability factor. * Values were significant for p < 0.001

**Figure 2:**
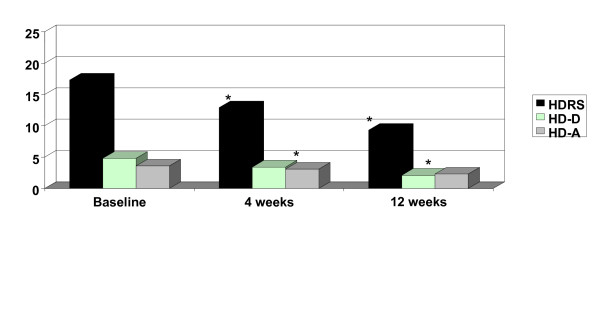
Significant decrease of Hamilton-D Rating Scale from baseline to 4 and 12 weeks. HDRS = Hamilton-D Rating Scale Total Score; HD-D = Ham-D Depressive factor. HD-A: Ham-D Anxiety Factor. * Values were significant for p < 0.001

Thirty-two and 23 patients attended the follow-up visits 4 and 12 weeks later, respectively. There was a substantial and highly significant (p < .001) decrease in the CGI score, HDRS total score, HDRS and SVARAD anxiety factors, HDRS and SVARAD core depression factors, and SVARAD anger/irritability factor (Table 2, Figures 1, 2). At the 12-week follow-up visit, most patients (82%) were rated as 'improved' or very much improved' on the Global Improvement item of the CGI. Similarly, 80% of patients experienced a reduction in HDRS total score of at least 35%. In most patients the HDRS total score decreased to levels indicating remission (0–7; 35%) or only mild depression (8–13; 50%).

**Table 2:** Changes in outcome measures over time

Outcome measure	Baseline (mean ± SD)	4 weeks (mean ± SD)	12 weeks (mean ± SD)
HDRS total score*	17.3 ± 3.3 (n = 35)	12.9 ± 3.7 (n = 32)	9.3 ± 3.9 (n = 20)
HDRS Core depression factor*	4.8 ± 1.4 (n = 35)	3.4 ± 1.5 (n = 32)	2.1 ± 1.3 (n = 20)
HDRS Anxiety factor*	3.6 ± 1.5 (n = 35)	3.1 ± 1.3 (n = 32)	2.3 ± 1.0 (n = 20)
SVARAD Core depression factor*	3.5 ± 1.2 (n = 35)	2.2 ± 1.2 (n = 32)	1.1 ± 1.0 (n = 23)
SVARAD Anxiety factor*	3.1 ± 1.3 (n = 35)	2.4 ± 1.2 (n = 32)	2.0 ± 1.5 (n = 23)
SVARAD Anger/irritability factor*	3.2 ± 1.5 (n = 35)	1.7 ± 1.2 (n = 32)	1.0 ± 0.8 (n = 23)

* p < 0.001

On a percentage basis, the improvement was most pronounced in anger/irritability, marked in depressed mood, and moderate in anxiety. The observed improvement remained highly significant (p < .001 for all outcome measures) when adjusting for either gender or age. Also, when we stratified patients according to the prescribed antidepressant (paroxetine, fluvoxamine, or citalopram) or anticonvulsant, the improvement was consistent across strata on all outcome measures. Suicidal ideation was present in 12 (34%), 5 (23%), and zero patients at baseline, 4 weeks, and 12 weeks, respectively. The change in scores over time on the HDRS suicide items was highly significant (p = 0.005).

## Conclusion

This study has several limitations, including the lack of a placebo control group and the absence of control groups treated with only an SSRI or only an anticonvulsant. The lack of a control group treated with only one active drug precludes firm conclusions about the advantages of combined treatment over monotherapy. Also, our decision to use various SSRIs and anticonvulsants rather than a single drug in each class might be criticised, although it more closely resembles daily clinical practice. Further, while dropout was very limited (9%) at 4 weeks, follow-up data were not available for 34% of patients at 12 weeks. Usually, drop-outs occur for reasons such as lack of efficacy, adverse effect, or improvement. Unfortunately, we did not systematically investigated dropouts and cannot tell how many of these patients were unsatisfied with treatment. However, even a sensitivity analysis assuming a worst-case scenario (i.e., all drop-outs due to inefficacy or adverse effects) would still yield a percentage of patients rated as 'improved' or 'much improved' on the CGI as high as 54%. Moreover, the outcome was not independently assessed, because the raters were involved in the treatment of each patient. However, most patients were seen by two clinicians who both participated in the completion of the rating scales, and this should have reduced the risk of assessment bias.

While these limitations suggest great caution in interpreting the results of the present study and dictate the need of a replication on a larger sample with a controlled design, we observed that most unipolar depressed outpatients with dysphoric mood improved quickly when treated with an SSRI combined with an anticonvulsant. The use of the SVARAD, an assessment instrument covering a broad range of psychopathological dimensions, enabled us to detect a rapid and marked improvement not only in the depressive and anxiety dimensions, but also in the anger/irritability dimension. The average percentage of improvement on the anger/irritability dimension was 69%, while the average percentage of improvement on the depressive and anxiety dimensions was 56% and 36% on the HDRS and 69% and 35% on the SVARAD, respectively. While the selection of patients with high pre-treatment levels of anger/irritability may partly account for the greater improvement observed in this dimension, these findings suggest that the HDRS is less well suited to document improvement in depressed patients with dysphoric mood, because it contains many items tapping the relatively less responsive anxiety symptoms, while it includes no items exploring the anger/irritability dimension.

These findings suggest that adding valproate and possibly other anticonvulsants to antidepressant medication might be a profitable strategy not only in bipolar depression, but also when dealing with unipolar depressed patients presenting with prominent symptoms of anger, irritability, and hostility. Although our patients were not assessed using a structured diagnostic interview, the diagnoses were made after a careful psychiatric examination, and were confirmed by a very experienced faculty psychiatrist who reviewed all clinical records. While apparent unipolar depression might turn out to be bipolar II disorder on careful questioning [26] and some bipolar patients are misdiagnosed as unipolar [27], the clinicians involved in the study had been instructed to inquire about a history of mood swings, hypomania or mania, and this precaution should have minimised, though not ruled out, the risk of misdiagnosis. Indeed, only two patients had a clinically relevant score on item 10 which taps symptoms of behavioural activation or mood elevation. Also, it should be noted that prominent dysphoria in a depressed patient should not be taken as synonymous with bipolar depression or mixed state derived from the combination of depressive and manic symptoms. Several studies have documented that anger, irritability, and hostility can be present in unipolar patients, while the Vienna school of psychopathology has even suggested that dysphoria should be regarded as a third mood quality separated from depression and mania [28]. In this view, a dimensional approach to diagnosis might help designing a pathogenesis-oriented diagnosis in the tradition of Kretschmer [29]. While nosological disputes are beyond the scope of this paper, the nosology of mixed states has several limitations that have been underlined in an extensive review [30].

The relationship between aggressive behaviour and reduced serotonergic activity in the central nervous system has led several authors to suggest serotonergic treatment for psychiatric conditions with prominent anger or aggression. Previous studies did not observe a preferential response to SSRIs compared to other antidepressants in depressed patients with hostility [9,31]. Unfortunately, the design of our study does not permit us to disentangle the relative contribution of SSRIs to improvement.

In our study, the large majority of patients (74%) received valproate in addition to serotonergic treatment. Only a few patients received carbamazepine (9%), while some were prescribed gabapentin (17%). Hence, our findings apply mostly to valproate. Whereas its efficacy in acute mania [32] and possibly in the long-term maintenance treatment of bipolar disorder [33] is supported by double-blind placebo controlled trials, there are no controlled trials on its efficacy in depression with dysphoric mood. Interestingly, there is no agreed definition of the term 'mood stabiliser'. Possibly, the use of a strictly categorical approach to diagnosis in clinical trials might obscure some pharmacological effects that are nevertheless clinically important in everyday practice, where one has to deal with those symptom clusters that are prominent in a given patient rather than with a categorical diagnosis. In this regard, it should be emphasised that we used valproate because to its documented effectiveness on symptoms of aggression and behavioural activation, rather than due to its still controversial mood stabilising properties.

As long suggested [34,15], overcoming the reductive strategy of prescribing psychotropic drugs based on a strict matching of drugs with diagnostic categories might permit to explore new strategies, such as treatments based on the specific pathophysiologic mechanisms of those symptom clusters or psychopathological dimensions that are relevant in each patient. A possible mechanism of action of some anticonvulsants in reducing hostile behaviours and anger could arise from their effects on the GABAergic system. For instance, valproate treatment results in increased GABA levels, inhibition of GABA catabolism, increase of postsynaptic GABA responses, and blockade of sodium channels implying a reduction of the glutamate response [34]. Carbamazepine has a tricyclic structure, with some GABAergic and antiglutamatergic effects, while gabapentin appears to have more subtle GABAergic effects such as increasing glial GABA release and action on GABA transporters [35]. Interestingly, irritability has been the most consistently differentiating symptom favouring valproate benefit in patients with disorders other than bipolar disorder [36,37].

In conclusion, depression with dysphoric mood may be regarded as a particular depressive condition that requires specific management. The proper identification of symptoms such as intense anger, irritability, hostility, and aggressiveness in a depressed patient is clinically important and has substantial implications for treatment. In spite of several limitations, this study showed a significant and rapid improvement not only in depressive and anxiety symptoms, but also in anger, irritability and hostility in unipolar depressive outpatients following a short course of treatment with a combination of an SSRI and an anticonvulsant, mostly valproate. If our results were confirmed, they would confirm the advantages of using a dimensional rather than a strictly categorical approach to psychopathological assessment and treatment of psychiatric conditions. Clearly, future studies with more robust methodology are needed to corroborate our findings.

## Abbreviations

DSM-IV Diagnostic and Statistical Manual of Mental Disorders

HDRS Hamilton Depression Rating Scale

CGI Clinical Global Improvement

SVARAD Scala per la VAlutazione RApida Dimensionale

SSRI Selective Serotonin Reuptake Inhibitor

## Competing interests

The author(s) declare that they have no competing interests.

## Authors' contributions

MP was involved in conception, designing, drafting and approving the final version. AP was involved in data analysis and interpretation, drafting and revising the final draft. AS, VO participated in the design of the study and data collection, and provided comment on the content of the manuscript. PM, LT, IC and MB revised the manuscript critically and contributed to the final draft.
